# Whole‐exome and HLA sequencing in Febrile infection‐related epilepsy syndrome

**DOI:** 10.1002/acn3.51062

**Published:** 2020-07-14

**Authors:** Ingo Helbig, Giulia Barcia, Manuela Pendziwiat, Shiva Ganesan, Stefanie H. Mueller, Katherine L. Helbig, Priya Vaidiswaran, Julie Xian, Peter D. Galer, Zaid Afawi, Nicola Specchio, Gerhard Kluger, Gregor Kuhlenbäumer, Silke Appenzeller, Michael Wittig, Uri Kramer, Andreas van Baalen, Rima Nabbout, FIRES Genetics Study Group

**Affiliations:** ^1^ Division of Neurology Children’s Hospital of Philadelphia Philadelphia Pennsylvania; ^2^ The Epilepsy NeuroGenetics Initiative (ENGIN) Children’s Hospital of Philadelphia Philadelphia Pennsylvania; ^3^ Department of Biomedical and Health Informatics Children’s Hospital of Philadelphia Philadelphia Pennsylvania; ^4^ Department of Neurology University of Pennsylvania Perelman School of Medicine Philadelphia Pennsylvania; ^5^ Reference Centre for Rare Epilepsies Department of Pediatric Neurology Necker Enfants Malades Hospital Paris Descartes University Paris France; ^6^ Laboratory of Translational Research for Neurological Disorders Institute Imagine Paris France; ^7^ Department of Genetics Necker Enfants Malades Hospital Imagine Institute Paris France; ^8^ Department of Neuropediatrics University Medical Center Schleswig‐Holstein Christian‐Albrechts University Kiel Germany; ^9^ Institute of Health Informatics UCL London UK; ^10^ Department of Neurology University Hospital Schleswig Holstein Kiel Germany; ^11^ Sackler School of Medicine Tel‐Aviv University Ramat Aviv Israel; ^12^ Department of Psychiatry Tel‐Aviv University Ramat Aviv Israel; ^13^ Erasmus University Medical Center Rotterdam The Netherlands; ^14^ Neurology Unit Department of Neuroscience Bambino Gesù Children's Hospital IRCCS Roma Italy; ^15^ Neuropediatric Clinic and Clinic for Neurorehabilitation Epilepsy Center for Children and Adolescents Vogtareuth Germany; ^16^ Research Institute Rehabilitation/Transition/Palliation PMU Salzburg Salzburg Austria; ^17^ Comprehensive Cancer Center Mainfranken University Hospital Würzburg Würzburg Germany; ^18^ Institute of Clinical Molecular Biology Christian‐Albrechts‐University of Kiel Kiel Germany; ^19^ Pediatric Neurology Center Dana‐Dwek Children's Hospital Tel Aviv Medical Center Tel Aviv Israel; ^20^ Sackler Faculty of Medicine Tel Aviv University Tel Aviv Israel; ^21^ Universite Paris Descartes ‐Sorbonne Paris City Imagine Institute Paris France

## Abstract

Febrile infection‐related epilepsy syndrome (FIRES) is a devastating epilepsy characterized by new‐onset refractory status epilepticus with a prior febrile infection. We performed exome sequencing in 50 individuals with FIRES, including 27 patient–parent trios and 23 single probands, none of whom had pathogenic variants in established genes for epilepsies or neurodevelopmental disorders. We also performed HLA sequencing in 29 individuals with FIRES and 529 controls, which failed to identify prominent HLA alleles. The genetic architecture of FIRES is substantially different from other developmental and epileptic encephalopathies, and the underlying etiology remains elusive, requiring novel approaches to identify the underlying causative factors.

## Introduction

Febrile infection‐related epilepsy syndrome (FIRES) is a severe epileptic encephalopathy with new‐onset super‐refractory status epilepticus that presents with a febrile illness prior to seizure onset.^1^ Status epilepticus in individuals with FIRES is highly refractory, and, even though a cytokine‐mediated mechanism has been proposed, the pathophysiology remains entirely unknown.^2^, ^3^ FIRES shares many clinical features with developmental and epileptic encephalopathies (DEE). Over the last two decades, genetic studies have identified the underlying cause of many previously poorly understood epilepsy syndromes, including disease‐causing *SCN1A* variants in up to 90% of individuals with Dravet Syndrome^4^ and disease‐causing *KCNT1* variants in a significant fraction of individuals with epilepsy with migrating focal seizures.^5^ Genetic testing has become a common diagnostic modality and is routinely performed in children and adults with DEE. The diagnostic yield is considered 15–20%^6^, ^7^ and reaches up to 50% in individuals with neonatal epileptic encephalopathies,^8^ largely due to de novo variants in genes encoding ion channels or synaptic proteins.

Ever since its initial description in 2010,^1^ the cause of Febrile Infection‐Related Epilepsy Syndrome (FIRES) has been a matter of debate. Given the absence of otherwise explanatory factors, two avenues of research have been pursued to better understand the underlying disease mechanism, including (1) the identification and delineation of a potentially dysregulated immune response and (2) genetic studies in parallel to genetic investigations in other nonlesional epilepsies. While studies into the disease mechanism have recently focused on a dysregulation of the IL1 response^9^ with reports suggesting a promising therapeutic effect of anakinra,^10^, ^11^, ^12^, ^13^ the primary cause leading to this response is unknown.

Given the suspicion of an underlying genetic cause, multiple studies over the last decade have assessed the role of genetic factors in FIRES (detailed in Supplementary Information). However, excluding individuals subsequently included in our study, we only identified less than 30 individuals with FIRES reported to have undergone sequencing for candidate genes, gene panel analysis, or whole exome sequencing in these studies. A subset of studies reported alleged genetic findings in FIRES. By current diagnostic criteria, however, none of the findings would be considered explanatory.

FIRES is extremely rare with less than 50 new cases in the United States every year. Accordingly, assembling patient cohorts is challenging. We therefore established an international study to perform exome sequencing in individuals with FIRES. We reasoned that, in parallel to DEE, autism, and other neurodevelopmental disorders, the genetic basis of FIRES can be identified through a modern next‐generation sequencing approach.

## Patients and Methods

### Subjects

We included a cohort of 50 individuals with FIRES from centers in Kiel, Germany (n = 30), Paris, France (n = 9), Israel (n = 7), Italy (n = 2), and the US (n = 2) for the current study. The diagnosis of FIRES was confirmed by two senior physicians (A.v.B., R.N.) based on consensus criteria,^14^ and standardized phenotyping was obtained for all individuals. The study was approved by the Institutional Review Board of the University of Kiel, Germany, Hôpital Necker‐Enfants Malades, Paris, France, and Children’s Hospital of Philadelphia.

### Exome sequencing and HLA sequencing

Exome sequencing was performed for patient–parent trios and singletons at four sites. Of these, 18 individuals were sequenced at Broad institute with Nextera Rapid Capture Exome kit through the Epi25 Project, 13 individuals at Cologne Center for Genomics with Agilent Technologies SureSelect V6 R2 kit, 11 individuals at Institut Imagine, Paris with Agilent Technologies 50 V5 kit, and 6 individuals with Truseq Exome Enrichment kit at the Institute of Clinical Molecular Biology (ICMB), Kiel. Molecular HLA typing was performed at ICMB using a targeted NGS method as previously reported.^15^

### Bioinformatic analysis for exome and HLA sequencing

Exome data were analyzed through a bioinformatic pipeline as previously described.^16^, ^17^ Analysis for pathogenic variants in genetic etiologies for human epilepsies was performed according to criteria of the American College of Medical Genetics and Genomics (ACMG).^18^ HLA imputation was performed using HLAscan, including 29 cases and 529 controls of European ancestry. Using PyHLA, significant HLA associations were tested under an additive allelic model,^19^ correcting for multiple testing using the Benjamini‐Hochberg FDR method.

### Selection of FIRES candidate genes

In trio whole‐exome data, we selected (a) de novo, X‐linked, and bi‐allelic variants absent in population databases. For both trio and singleton exome data, we further identified (b) ClinVar pathogenic variants,^20^ (c) nonpopulation variants in 101 epilepsy‐related genes, (d) Protein Truncating Variants (PTV) in genes resistant to loss‐of‐function variation (pLI score > 0.95),^21^ and (e) deleterious missense variants (CADD score > 20) within constrained coding regions (CCR > 95th percentile).^22^ The selection strategy for these candidate genes is further outlined in the Supplementary Information.

## Results

### Spectrum of clinical features

Our cohort included 17 females and 33 males (clinical features are shown in Table S1). In brief, three individuals (F3, F44, and F51) had prior febrile seizures, but none of the individuals had epilepsy diagnosed prior to the onset of FIRES. Median age of onset of the acute phase was 6 years (range 2–15 years). EEGs performed in the acute phase showed epileptiform activity in 41/50, mostly multifocal discharges arising bilaterally from the frontal and temporal regions. Developmental outcome was documented in 49/50 individuals, including 11/49 with mild, 18/49 with moderate, and 13/49 with severe intellectual disability. Seven individuals had resolution of FIRES without intellectual deficits. 44/47 individuals with documented development prior to seizure onset did not have developmental concerns, the remaining three individuals had anxiety and obsessive‐compulsive disorder (F11), attention deficit hyperactivity disorder (ADHD, F14), and language delay (F43).

### Exome sequencing

#### Known genetic etiologies in human epilepsies

None of the 50 individuals had pathogenic or likely pathogenic variants in known genetic etiologies for human epilepsies. The 27 patient–parent trios were further analyzed for de novo, X‐linked, and bi‐allelic variants absent in population databases (Table 1). No gene was found to be affected by a de novo variant or bi‐allelic variants in two or more individuals. The only known variant in a gene related to human epilepsies was a previously reported de novo c.G1117A (p.E373K) variant in *DNM1* in F26.^23^ However, as this variant in *DNM1* occurred outside the typical mutation cluster and the presentation was incompatible with the typical phenotype, it remained of uncertain significance. We next analyzed a virtual panel of 101 curated epilepsy‐related genes that are typically analyzed in a diagnostic context (Table S2). This analysis identified a total of seven variants absent in population databases, including two variants in *KCNQ3* (F38 and F28) and one variant in *SCN2A* (F29). The *KCNQ3* variant in F38 was paternally inherited and parental testing was not available for F28. Both these *KCNQ3* variants occurred in the C‐terminal end, where population variants in this gene are common and hence, a profound effect on protein function is unlikely. The variant c.A2851G (p.M951V) in *SCN2A* occurred close to the selectivity filter, paralogous to *SCN1A* variants observed in Dravet Syndrome.^24^ However, the variant in F29 was inherited from an unaffected father and is not considered explanatory for the individual’s epilepsy. All other nonpopulation variants in epilepsy genes occurred in genes incompatible with the FIRES phenotype. The analysis of ClinVar pathogenic variants identified a reported pathogenic variant in *FOXP2* (p.Q175delinsQQQQQ) in F47, a known genetic etiology for speech disorders. As this variant was inherited from an unaffected father, we consider it noncontributory to the FIRES phenotype.

**Table 1:** Candidate variants in FIRES.

ID	Test	Inheritance	Gene	Variant (c./p.)	Protein name	Transcripts
Variants with monogenic inheritance						
F17	Trio	de novo	*NPY2R*	c.A52G;p.K18E	Neuropeptide y receptor y2	NM_000910.3
F23	Trio	de novo	*MYO1D*	c.A1348G;p.K450E	Myosin ID	NM_001303279.1
F23	Trio	de novo	*UNC50*	c.582dupA;p.G194fs	Unc‐50 Inner nuclear membrane RNA binding protein	NM_001330353.1
F23	Trio	de novo	*SPICE1*	c.G1540C;p.D514H	Spindle‐ and centriole‐associated protein 1	NM_001331078.1
F32	Trio	de novo	*NAV1*	c.G982A;p.G328S	Neuron navigator 1	NM_020443.4
F7	Trio	de novo	*LRIF1*	c.C1585T;p.Q529X	Ligand‐dependent nuclear receptor‐interacting factor 1	NM_018372.3
F8	Trio	Compound	*UNC79*	c.G4087T;p.V1363L c.C4654A;p.L1552I	Unc79 homolog, NALCN channel complex subunit	NM_001346218.1
F29	Trio	de novo	*KDM2B*	c.G3172A;p.V1058I	Lysine‐specific demethylase 2b	NM_001005366.1
F32	Trio	Compound	*KIAA0586*	c.137delG;p.R46fs c.1793_1794del; p.E598fs	KIAA0586 protein	NM_001244191.1
F26	Single	de novo^1^	*DNM1*	c.G1117A;p.E373K	Dynamin 1	NM_004408.2
ClinVar pathogenic variants for neurological disorders						
F27	Single	Unknown	*SPG7*	c.C1369T;p.R457X	Paraplegin (Hereditary spastic paraplegia)	NM_003119.3
F47	Trio	Paternal	*FOXP2*	c.525_526insCAGCAG CAACAA; p.Q175delinsQQQQQ	Forkhead box protein p2 (Speech‐language disorder‐1)	NM_148900.3
F17	Trio	Maternal	*POLG*	c.G1399A;p.A467T	DNA Polymerase Gamma, Catalytic Subunit (Leigh Syndrome)	NM_001126131.1
F30	Trio	Paternal	*CAPN3*	c.549delA;p.P183fs	Calpain 3 (Limb‐girdle muscular dystrophy)	NM_000070.2
Variants in genetic etiologies for epilepsy absent in gnomAD database						
F35	Single	Unknown	*GNAO1*	c.A425C;p.N142T	G Protein Subunit Alpha O1	NM_020988.2
F38	Single	Homozygous	*LGI1*	c.A615T;p.E205D	Leucine‐rich gene, glioma‐inactivated, 1	NM_001308275.1
F38	Single	Paternal	*KCNQ3*	c.G1898A;p.G633E	Potassium Voltage‐Gated Channel Subfamily Q Member 3	NM_004519.3
F38	Single	Unknown	*ARX*	c.G503A;p.S168N	Aristaless‐related homeobox	NM_139058.2
F27	Single	Unknown	*ALG13*	c.C905T;p.A302V	UDP‐N‐Acetylglucosaminyl‐transferase	NM_001257230.1
F28	Single	Unknown	*KCNQ3*	c.G1854A;p.M618I	Potassium Voltage‐Gated Channel Subfamily Q Member 3	NM_004519.3
F29	Trio	Paternal	*SCN2A*	c.A2851G;p.M951V	Sodium channel, voltage‐gated, type ii, alpha subunit	NM_001040143.1

^1^ Previously published as de novo^23^.

#### Candidate variants and Copy Number Variation (CNV) analysis

We identified de novo variants absent in population databases in *NPY2R*, *MYO1D*, *UNC50*, *SPICE1*, *NAV1*, and *LRIF1*. None of these genetic etiologies are established in neurodevelopmental disorders or epilepsy, but occur in brain‐expressed genes intolerant to mutation, including genes implicated in brain development (*NPY2R*, *UNC50*, *NAV1,* Supplementary Information). We further assessed potential candidate variants by identifying protein‐truncating variants in genes resistant to loss‐of‐function variation and missense variants in genomic regions devoid of variation. We identified a total of seven protein truncating variants and 10 missense variants (Table 2). None of the implicated genes are established causes for neurological disease. Analysis of copy number variations from exome sequences did not identify pathogenic deletions or duplications (Supplementary Information).

**Table 2:** Qualifying protein‐truncating variants and missense variants in FIRES.

ID	Test	Inheritance	Gene	Variant (c./p.)	Protein name	Transcripts
Protein‐truncating variants (PTV) with pLI > 0.95						
F41	Single	Unknown	*TBX1*	c.1065_1066del; p.P355fs	T‐Box Transcription Factor 1	NM_080646.1
F33	Single	Unknown	*GNAS*	c.G29A;p.W10X	Guanine Nucleotide Binding Protein, Alpha Stimulating Activity Polypeptide 1	NM_016592.3
F34	Single	Unknown	*WWP2*	c.G403T;p.G135X	NEDD4‐Like E3 Ubiquitin‐Protein Ligase WWP2	NM_001270454.1
F51	Trio	Paternal	*JAKMIP1*	c.G2053T;p.E685X	Janus Kinase and Microtubule Interacting Protein 1	NM_001099433.1
F1	Trio	Maternal	*HSP90AB1*	c.2172_2176del; p.D724fs	Heat Shock Protein 90kDa Alpha (Cytosolic), Class B Member 1	NM_001271969.1
F30	Trio	Maternal	*BRD1*	c.1412delA; p.D471fs	Bromodomain‐containing protein 1	NM_001349940.1
F30	Trio	Maternal	*ITGB8*	c.C208T;p.Q70X	Integrin, beta‐8	NM_002214.2
Missense with phred‐scaled CADD score > 20 and conserved coding region (CCR) percentile > 90						
F35	Single	Unknown	*ABCB6*	c.A2175C;p.K725N	Phosphatidylinositol glycan anchor biosynthesis class X protein	NM_001349828.1
F35	Single	Unknown	*MGAT4D*	c.T919A;p.F307I	Mannosyl (Alpha‐1,3‐)‐Glycoprotein Beta‐1,4‐N‐Acetylglucosaminyltransferase Family, Member D	NM_001277353.1
F38	Single	Unknown	*DIP2C*	c.C3183G;p.H1061Q	Disco‐interacting protein 2 homolog C	NM_014974.2
F22	Single	Unknown	*NDUFAB1*	c.A1C;p.M1L	NADH‐ubiquinone oxidoreductase 1 alpha/beta subcomplex, 1	NM_005003.2
F27	Single	Unknown	*BCAS4*	c.G619A;p.A207T	Breast carcinoma amplified sequence 4	NM_017843.4
F46	Single	Unknown	*RC3H2*	c.G3164C;p.R1055T	Ring finger and CCCH‐type zinc finger domains‐containing protein 2	NM_001100588.1
F31	Trio	Maternal	*CDKN2AIP*	c.C1708T;p.P570S	Cyclin‐dependent kinase inhibitor 2A‐interacting protein	NM_017632.3
F48	Trio	Maternal	*HIST2H2AB*	c.C222G;p.N74K	Histone gene cluster 2, H2A histone family, Member B	NM_175065.2
F7	Trio	Paternal	*GHRH*	c.A164C;p.Q55P	Growth hormone‐releasing hormone	NM_001184731.2
F42	Single	Unknown	*SLC25A1*	c.526 + 1G>A	Solute Carrier Family 25 (Mitochondrial Carrier; Citrate Transporter), Member 1	NM_005984.4

### HLA sequencing

We performed HLA sequencing in 29 individuals and compared allelic associations with 529 population controls. We did not identify a prominent HLA allele but found tentative associations with HLA‐C*07:01 [OR 8.7, 95% CI 3.55–21.30], HLA‐A*02:05 [OR 12.99, 95% CI 3.56–47.39], and HLA*A: 03:01 [OR 0.1, 95% CI 0.01–0.77]. Given the lack of a consistent HLA signature, we consider these results inconclusive. None of the identified HLA associations have previously been reported in other disorders.

## Discussion

In our study, we aimed to identify the genetic basis of FIRES using whole exome sequencing, reasoning that the clinical features are related to developmental and epileptic encephalopathies (DEE) where genetic causes are routinely identified. None of the individuals with FIRES had explanatory variants in known genetic etiologies for epilepsies or neurodevelopmental disorders. Even though we identified several variants of uncertain significance in known epilepsy‐related genes and potential candidate genes, none of the identified variants were considered disease‐causing.

In our study, we do not observe the same rate of pathogenic variants in established epilepsy genes that we would expect in an equally‐sized cohort of individuals with DEE, such as Infantile Spasms or Lennox‐Gastaut Syndrome where the diagnostic rate is 15% or higher. Assuming a comparable diagnostic rate for FIRES, we would have expected at least three individuals with pathogenic variants, allowing for the conclusion that the rate of pathogenic or likely pathogenic variants in known epilepsy genes in FIRES is low (95% CI 0–0.09 for variant frequency in 0/50 individuals; *P* = 0.006 for null probability of 0.15, see Supplementary Information). Status epilepticus in FIRES is preceded by a mild febrile illness,^14^, ^25^ suggesting a possible immune or inflammatory mechanism. None of the candidate genes identified in our study are implicated in human immunological disorders, and HLA sequencing failed to identify a strong disease association. Tentative associations with HLA‐C*07:01, HLA‐A*02:05, and HLA‐A*03:01 were nonexplanatory. We cannot exclude that inherited variants, including the rare variants identified in our analysis, may contribute to the risk of FIRES. However, given the low frequency of FIRES and the small sample size, we are currently unable to make a definite statement about the role of both rare and common genetic variants in the etiology of FIRES.

Our study highlights that known genetic causes for epilepsy are not common in FIRES, which may be important in a diagnostic setting. Even though individuals with FIRES will continue to undergo a genetic work‐up, our data does not provide a rationale for prioritizing genetic studies over other diagnostic modalities. Likewise, our data advocates against withholding specific anti‐seizure medications based on genetic considerations, including a reluctance to use sodium channel blockers or valproic acid due to concerns for an underlying sodium channelopathy or *POLG*‐related disorder. Examining non‐genetic contributors or genetic mechanisms not covered by exome sequencing will be critical for future research, including the analysis of repeat expansions or non‐coding regions that are increasingly recognized in neurological disorders.

## FIRES Genetics Study Group

Joel Fluss^1^, Andre Franke^2^, Martin Häusler^3^, Wolfgang Lieb^4^, Eiko Nausch^5^, Annika Rademacher^5^, Malte Ziemann^6^

^1^Pediatric Neurology Unit, Geneva Children's Hospital, Genève, Switzerland

^2^Institute of Clinical Molecular Biology, Christian‐Albrechts‐University of Kiel, Kiel, Germany

^3^Division of Neuropediatrics and Social Pediatrics, Department of Pediatrics, University Hospital, Aachen, Germany

^4^Institute of Epidemiology and Biobank PopGen, Kiel University, Kiel, Germany

^5^Department of Neuropediatrics, University Medical Center Schleswig‐Holstein, Christian Albrechts University Kiel, Germany

^6^Institute of Transfusion Medicine, University Hospital of Schleswig‐Holstein, Lübeck‐ Kiel, Germany

## Author Contributions

I.H., A.v.B., and R.N. conceptualized and designed the study. I.H., U.K., Z.A., N.S., A.v.B., and R.N. contributed clinical patients to the study. M.P., S.A., and G.K. performed genetic studies including Sanger sequencing, G.B., M.P., S.A., S.G., K.L.H. and J.X. performed exome data analysis, S.H.M. and M.W. performed analysis of HLA sequencing data. I.H., P.V., G.K., U.K., A.v.B., and R.N. performed phenotype analysis. I.H., S.G., and P.D.G. drafted the publication, which was reviewed and edited by all authors.

## Conflict of Interest

Nothing to report.

## Supporting information


**Table S1.** Clinical characteristics in individuals with FIRES.
**Table S2.** Gene list for virtual epilepsy gene panel.
**Table S3.** Comparison of virtual gene panel content to epilepsy gene panel studies in the literature.
**Table S4.** Significant associations between HLA alleles and FIRES.
