# Supplementary material for: Whole‐exome and HLA sequencing in Febrile infection‐related epilepsy syndrome
Source: Ann Clin Transl Neurol. 2020 Jul 14;7(8):1429–35. doi: 10.1002/acn3.51062 (PMC7448193; doi:10.1002/acn3.51062)
Supplement: Supplementary file 1 — Table S1. Clinical characteristics in individuals with FIRES. Table S2. Gene list for virtual epilepsy gene panel. Table S3. Comparison of virtual gene panel content to epilepsy gene panel studies in the literature. Table S4. Significant associations between HLA alleles and FIRES. [file ACN3-7-1429-s001.docx]

# **Whole-exome and HLA sequencing in Febrile Infection-Related Epilepsy Syndrome**

## **Supplementary Information**

**Overview of prior studies investigating a genetic etiology for FIRES**

In a literature search for genetic studies into the potential cause of Febrile Infection-Related Epilepsy Syndrome, we identified 12 studies between November 2011 and February 2020 utilizing the PubMed search terms "Febrile-infection related epilepsy syndrome" AND ("gene" or "genetic" or "exome"). In addition, we included a single study that we were aware of that had 12 individuals with FIRES^1^ who would not have been retrievable through a PubMed search. In total, excluding individuals subsequently included in our study, we identified less than 30 individuals with FIRES reported to have undergone sequencing for candidate genes, gene panel analysis, or whole exome sequencing. A subset of studies reported alleged genetic findings in FIRES. However, by diagnostic criteria, none of the findings would be considered explanatory.

An early study by Specchio and collaborators in 2011 reported an 8 month-old girl with a *de novo* variant in PCDH19 with a sudden onset of frequent partial seizures that evolved to refractory status epilepticus after a rotavirus gastroenteritis.^2^ However, as acknowledged by the authors, their patient was relatively young, and, while the phenotype somewhat overlapped with FIRES, the authors provided a useful guide to differentiate FIRES from the entity that has since become known as *PCDH19* Epilepsy,^3^ including fever at onset which is typically present in *PCDH19* Epilepsy and always absent in FIRES. Since the initial report, we and others have demonstrated that disease-causing variants in *PCDH19* are not a common cause of FIRES, and no other individuals with FIRES and variants in *PCDH19* have been reported since.

The study by Kambouris and collaborators in 2010 identified a bi-allelic variant in *SCN10A* in an individual with FIRES.^4^ However, *SCN10A* is not established as a disease gene according to ClinGen variants, and no additional reports have supported a role for this genetic etiology since.^5^

The study by Saitoh and collaborators in 2016 studied 19 individuals with FIRES.^6^ However, this study only investigated common polymorphisms and did not include full sequencing of possible disease genes.

A single individual with a *de novo* variant in *DNM1* was reported in our prior publication on the spectrum of *DNM1*-related phenotypes^7^ and is also included in our current study. However, the phenotype of this individual differs significantly from the typical *DNM1* phenotype, which often presents with onset of infantile spasms in the first year of life progressing to intractable Lennox-Gastaut Syndrome. In addition, the variant location stood apart significantly from the typical variant clusters seen in *DNM1* that predominantly occur in the GTPase and middle domain, which is thought to mediate the presumed dominant-negative mechanism of disease-causing variants in *DNM1* encephalopathy.^8^

|  | **Study** | **Individuals with FIRES** | **Comments** |  |
| --- | --- | --- | --- | --- |
|  | Specchio et al., 2011 | 1 | Individual with *de novo* variant in *PCDH19*, onset at 8 months |  |
|  | Appenzeller et al., 2012 | 12 | Absence of disease-causing variants in *SCN1A*, *POLG*, and *PCDH19* (all individuals included in current study) |  |
|  | Carranza Rojo et al., 2012 | 10 | Absence of disease-causing variants in *SCN1A* |  |
|  | Carvill et al., 2013 | 12 | Absence of disease causing variants in 19 known and 46 candidate epilepsy-related genes |  |
|  | Von Spiczak et al., 2014 | 1 | Individual with *de novo* variant in *DNM1* (included and discussed in current study) |  |
|  | Saitoh et al., 2016 | 19 | Analysis of common polymorphisms only |  |
|  | Kambouris et al., 2016 | 1 | Single individual with bi-allelic variant in *SCN10A*, limited gene validity |  |
|  | Rochtus et al., 2020 | 5 | Negative exome sequencing |  |
|  | **Current study** | **50** | **Negative exome sequencing including 27 patient-parent trios** |  |
|  |  |  |  |  |

**Table.** Overview of genetic studies in Febrile Infection-Related Epilepsy Syndrome (FIRES). Apart from the current study, less than 30 individuals have been reported with candidate gene sequencing, gene panel analysis, or whole exome sequencing. However, a genetic etiology has been systematically assessed in a variety of studies over the last decade by multiple research groups.

**Selection of candidate genes for virtual gene panel of 101 genes**

For the current study, we identified a subset of 101 genes that we analyzed as a virtual gene panel, e.g. exome data was selectively filtered for variants in these genes. The panel of 101 genes was selected from a curated gene panel at Children’s Hospital of Philadelphia^9^ and updated according to the state of the literature in 2020. Genes added to the panel include 17 genes (*DYRK1A, SMC1A*, *NPRL3*, *NEXMIF*, *CHRNA4*, *CHRNB2*, *SCN3A*, MTOR*, KCNC1, GRIN2D, CACNA1E, CACNA1G, KCNQ5*, *KCNT1*, *POLG*, *MECP2*, and *NPRL2)*. **Supplementary Table 2** lists all genes included on the virtual gene panel in this study.

Gene panel testing is typically considered the first line test for many epilepsies with a presumed genetic etiology, and the majority of genetic causes in childhood epilepsies are identified in a subset of genes. **Supplementary Table 3** compares the 101 genes of our virtual gene panel and the total number of individuals with disease causing variants in each gene in a joint analysis of three large gene panel studies in the epilepsies that collectively assessed more than 20,000 individuals.^10-12^ The number of individuals in these studies is listed and the rank of each genetic cause with regards to the total number of individuals among all 707 genetic etiologies was assessed, including all genes that were present on at least one gene panel in any of the three studies. Five genes on our virtual gene panel were not assessed in any of the three studies as these causes for genetic epilepsies had only been identified recently (*CACNA1E*, *CACNA1G*, *KCNQ5*, *NPRL2*, and *PIGT*). Out of the top 25 genes assessed in the gene panel studies, only a single gene was not included in our virtual panel (*CACNA1H*) as this gene is no longer considered a genetic cause for epilepsy.^5, 13^

For the selection of variants from our virtual panel of 101 genes shown in **Table 1**, we filtered exome sequencing data for variants that were absent in population databases, relying on the gnomAD database that includes 125,748 exome sequences and 15,708 whole-genome sequences from unrelated individuals sequenced as part of various disease-specific and population genetic studies.^14^ Amongst the variants selected for **Table 1**, we purposefully excluded single variants of uncertain significance in disorders with recessive inheritance. None of the individuals included in our cohort had bi-allelic variants (e.g. homozygous or compound heterozygous variants) or single variants classified as pathogenic or likely pathogenic in genes listed as recessive in **Supplementary Table 1**.

**Secondary genetic analysis for low-frequency population variants and copy number variants**

As some recessive disorders can be caused by variants with a low population frequency, we repeated our analysis for recessive variants with a population frequency cut-off of 0.01, which did not reveal individuals with bi-allelic variants. We also performed analysis for copy number variants from exome data using a modified ExomeDepth workflow.^15^ We did not identify individuals with pathogenic or likely pathogenic copy number variants.

**Selection strategy for candidate genes – monogenic inheritance**

Given the negative result of our study, we used two complementary strategies to identify potential candidate genes, which we defined as genes without an established gene-disease relationship. First, we identified variants with monogenic inheritance, including *de novo* and bi-allelic (recessive and compound heterozygous) variants. Second, we identified variants that either occurred as protein-truncating variants in genes with few population variants or missense variants in conserved regions of the genome (see below). The identified monogenic variants are briefly discussed below.

Individual F17 was found to have a *de novo NPY2R* c.A52G;(p.K18E) variant. The *NPY2R* encodes neuropeptide Y receptor Y2, which belongs to the group of G protein-coupled receptors present in the brain and sympathetic neurons for neuropeptide Y (NPY), a small neuropeptide involved in various processes in both the central and peripheral nervous system. A connection between neuropeptide Y and epilepsy is not established, but dysfunction of NPY has been suggested to be involved in cocaine dependence.^16, 17^ As a candidate gene for FIRES, dysfunction of NPY signaling may hint towards novel mechanisms outside the established models for pathogenicity in human epilepsies.

Individual F23 was found to carry a *de novo MYO1D* c.A1348G;(p.K450E) variant. The *MYO1D* gene encodes Myosin 1D, an unconventional myosin expressed in myelinating oligodendrocytes. *MYO1D* is considered to be involved in the generation and maintenance of CNS myelin,^18^ and there is a tentative link between genetic alteration in *MYO1D* and autism based on at least one individual with a partial deletion in this gene.^19^ Given the effect on CNS maturation, dysfunction of Myosin 1D may render the CNS susceptible to acute neurological events such as status epilepticus, even though a clear mechanism is not established to date.

The same individual was also found to have a *de novo* c.582dupA;(p.G194fs) variant in *UNC50*. Bi-allelic variants in *UNC50* encode a trafficking protein required for the assembly of nicotinic acetylcholine receptors.^20^ Bi-allelic loss-of-function variants in *UNC50* have been shown to cause arthrogryposis with asymptomatic heterozygous carriers.^21^ Given the strong body of evidence linking dysfunction of nicotinic acetylcholine receptors to epilepsies, particularly the familial nocturnal frontal lobe epilepsies,^22-24^ a similar mechanism may predispose individuals with *de novo* loss of function variants for seizures.

Individual F23 was also found to carry a third *de novo* variant, c.G1540C;(p.D514H) in *SPICE1*. The *SPICE1* gene encodes spindle- and centriole-associated protein 1, which is involved in spindle formation during mitosis, involved in centriole duplication and bipolar spindle formation.^25^ An association between *SPICE1* and neurological disorders has not been established yet, and it remains unclear how dysfunction of the *SPICE1* protein may result in epilepsy.

Individual F32 was found to have a *de novo NAV1* c.G982A;(p.G328S) variant. The *NAV1* gene encodes the neuron navigator 1 protein, which belongs to a brain-expressed family of genes involved in axon guidance.^26, 27^ While there is no established connection between this gene family and the epilepsies, dysfunction of *NAV1* in axon guidance may result in subtle maturational and connectivity defects that predispose an individual to a sudden onset of seizures similar to *PCDH19*-related epilepsies.^28^

Individual F7 was found to carry a protein-truncating c.C1585T;(p.Q529X) variant in *LRIF1*. The *LRIF1* gene encodes ligand-dependent nuclear receptor-interacting factor 1, a protein involved in accurate chromosome segregation during mitosis.^29^ A link between *LRIF1* and neurological disorders has not yet been established.

Individual F8 was found to carry bi-allelic variants in *UNC79* [c.G4087T;(p.V1363L) and c.C4654A;(p.L1552I)]. *UNC79* encodes a subunit of the *NALCN* channel complex subunit.^30^ Pathogenic variants in *NALCN* cause congenital contractures of the limbs and face, hypotonia, developmental delay (CLIFAHDD), ^31^ infantile hypotonia with psychomotor retardation, and characteristic facies type 1.^32^ In addition, pathogenic variants in the *UNC80* gene, which constitutes a further subunit of the UNC79-UNC80-NALCN channel complex, have recently been found to cause autosomal recessive severe infantile encephalopathy.^33, 34^ *UNC79* functions as an accessory subunit of *NALCN*, encoding a nonselective cation channel involved in the regulation of neuronal resting membrane potential and excitability.^35^

Individual F29 had a *de novo* c.G3172A;(p.V1058I) variant in *KDM2B*. The *KDM2B* gene encodes lysine-specific demethylase 2b. While the precise function of the protein in the CNS remains to be clarified, a recent study suggests that *KDM2B* is important in the differentiation and migration of cortical projection neurons.^36^ Accordingly, a mild dysfunction due to a *de novo* variant may result in a predisposition to seizures.

Individual F32 was found to carry bi-allelic protein truncating variants in *KIAA0586* [c.137delG;(p.R46fs), c.1793_1794del; (p.E598fs)]. The *KIAA0586* protein is ubiquitously expressed during fetal development and postnatally and has an unknown function. Homozygous or compound heterozygous mutations in the *KIAA0586* gene are known to cause Joubert Syndrome and short-rib thoracic dysplasia 14 with polydactyly.^37, 38^ The FIRES phenotype is clearly distinct from these presentations and, given some degree of variable expressivity and the presence of homozygous protein-truncating variant in unaffected individuals,^39^ the role of these bi-allelic variants in F32 remains unclear. If confirmed in other individuals with FIRES, it could be potentially postulated the epilepsies may represent yet another phenotypic presentation of bi-allelic *KIAA0586* protein-truncation variants.

Individual F26 was found to carry a *de novo DNM1* c.G1117A;(p.E373K) variant that has been previously reported and discussed above.^7^

**Selection strategy for candidate genes – protein-truncating variants and missense variants in conserved genomic regions**

To expand potential candidate variants beyond those inherited in a monogenic fashion, we identified protein truncating variants (PTV) in genes that are intolerant to loss of function variants in the population. This strategy also allowed us to assess variants from singleton exomes where monogenic inheritance often could not be established due to the absence of parental genetic information (**Table 2**). The probability of loss-of-function intolerance (pLI) indicates the probability that a gene is intolerant due to a Loss of Function (LoF) mutation.^40, 41^ Many of the genes with a high pLI such as *SCN1A* are known disease genes for neurological disorders. Protein-truncating variants in genes with a pLI of 0.95 or higher are rare in the population, and the presence of such variants may hint at their potential role in the patient’s disease. Given that none of the genes have previously been implicated, we decided to list these variants in **Table 2** as potential candidates without discussing their potential role in detail.

In addition to genes that have fewer loss of function variants than would be expected in the population, there are discrete genomic coding regions that are devoid of missense variation, even in genes that may not have a high pLI. These genomic regions are thought to be essential for the function of critical genes. The discrete genomic regions are referred to as conserved coding regions (CCR) and can be ranked as percentiles depending on their level of conservation.^42^ Consequently, missense variants can be annotated whether they occur in these regions. We identified missense variants in CCRs above the 90^th^ percentile, i.e. missense variants in the top 10% most conserved coding regions of the human genome (**Table 2, lower panel**). Within these regions, we searched for variants that are thought to have a strong impact on protein function based on bioinformatic prediction. As a prediction tool, we used the CADD score, a score that ranks genetic variants throughout the human genome and has been shown to be superior to other predictors.^43^ We use the phred-scaled CADD score with a cut-off of 20, identifying variants among the top 1% of predicted deleterious variants in the human genome. Taken together, filtering for CCR and CADD scores allows us to identify predicted deleterious variants in coding regions of the human genome that are highly intolerant to variation. Given that none of the variants occur in genes associated with disease, we have listed these variants in **Table 2, lower panel** as potential candidates.

**Power calculations and underlying assumptions for variant frequency**

Even though our cohort is relatively small, we wanted to assess whether the absence of pathogenic variants in known epilepsy genes through our virtual panel analysis allows us to draw conclusions about the frequency of genetic causes in FIRES more generally. In an equally-sized cohort of individuals with Infantile Spasms or Lennox-Gastaut Syndrome, a genetic etiology would be identified in 15% of individuals, though some studies report significantly higher frequencies. Based on the conservative estimate of 15%, we assessed whether a similar frequency in FIRES would be compatible with our findings in 50 individuals with FIRES. Assuming a frequency of 0.15, the 95% confidence interval ranges from 0.07-0.29. Accordingly, at the lower range of the confidence interval, 0.07 * 50 (3.5) individuals with pathogenic variants in known epilepsy genes would have been expected. This led us to the conclusion that we would have expected at least three individuals with pathogenic variants in known epilepsy genes. The frequency of 0/50 individuals is compatible with a 95% CI of 0-0.09, suggesting that while we cannot exclude that some individuals with FIRES have pathogenic variants in known epilepsy genes, the frequency is unlikely higher than 9%, a significant difference from what is usually found in a diagnostic context in childhood epilepsies. The analyses were performed using the R Statistical Framework.^44^

**Supplementary References**

1. Carvill GL, Heavin SB, Yendle SC, et al. Targeted resequencing in epileptic encephalopathies identifies de novo mutations in CHD2 and SYNGAP1. Nat Genet. 2013 Jul;45(7):825-30.

2. Specchio N, Fusco L, Vigevano F. Acute-onset epilepsy triggered by fever mimicking FIRES (febrile infection-related epilepsy syndrome): the role of protocadherin 19 (PCDH19) gene mutation. Epilepsia. 2011 Nov;52(11):e172-5.

3. Smith L, Singhal N, El Achkar CM, et al. PCDH19-related epilepsy is associated with a broad neurodevelopmental spectrum. Epilepsia. 2018 Mar;59(3):679-89.

4. Kambouris M, Thevenon J, Soldatos A, et al. Biallelic SCN10A mutations in neuromuscular disease and epileptic encephalopathy. Ann Clin Transl Neurol. 2017 Jan;4(1):26-35.

5. Helbig I, Riggs ER, Barry CA, et al. The ClinGen Epilepsy Gene Curation Expert Panel-Bridging the divide between clinical domain knowledge and formal gene curation criteria. Hum Mutat. 2018 Nov;39(11):1476-84.

6. Saitoh M, Kobayashi K, Ohmori I, et al. Cytokine-related and sodium channel polymorphism as candidate predisposing factors for childhood encephalopathy FIRES/AERRPS. J Neurol Sci. 2016 Sep 15;368:272-6.

7. von Spiczak S, Helbig KL, Shinde DN, et al. DNM1 encephalopathy: A new disease of vesicle fission. Neurology. 2017 Jul 25;89(4):385-94.

8. Dhindsa RS, Bradrick SS, Yao X, et al. Epileptic encephalopathy-causing mutations in DNM1 impair synaptic vesicle endocytosis. Neurol Genet. 2015 Jun;1(1):e4.

9. Balciuniene J, DeChene ET, Akgumus G, et al. Use of a Dynamic Genetic Testing Approach for Childhood-Onset Epilepsy. JAMA Netw Open. 2019 Apr 5;2(4):e192129.

10. Heyne HO, Artomov M, Battke F, et al. Targeted gene sequencing in 6994 individuals with neurodevelopmental disorder with epilepsy. Genet Med. 2019 Nov;21(11):2496-503.

11. Lindy AS, Stosser MB, Butler E, et al. Diagnostic outcomes for genetic testing of 70 genes in 8565 patients with epilepsy and neurodevelopmental disorders. Epilepsia. 2018 May;59(5):1062-71.

12. Truty R, Patil N, Sankar R, et al. Possible precision medicine implications from genetic testing using combined detection of sequence and intragenic copy number variants in a large cohort with childhood epilepsy. Epilepsia Open. 2019 Sep;4(3):397-408.

13. Calhoun JD, Huffman AM, Bellinski I, et al. CACNA1H variants are not a cause of monogenic epilepsy. Hum Mutat. 2020 Mar 30.

14. Karczewski KJ, Francioli LC, Tiao G, et al. Variation across 141,456 human exomes and genomes reveals the spectrum of loss-of-function intolerance across human protein-coding genes. bioRxiv. 2019:531210.

15. Rajagopalan R, Murrell JR, Luo M, Conlin LK. A highly sensitive and specific workflow for detecting rare copy-number variants from exome sequencing data. Genome Med. 2020 Jan 30;12(1):14.

16. Hwang YG, Lee HS. Neuropeptide Y (NPY) or cocaine- and amphetamine-regulated transcript (CART) fiber innervation on central and medial amygdaloid neurons that project to the locus coeruleus and dorsal raphe in the rat. Brain Res. 2018 Jun 15;1689:75-88.

17. Goncalves J, Martins J, Baptista S, Ambrosio AF, Silva AP. Effects of drugs of abuse on the central neuropeptide Y system. Addict Biol. 2016 Jul;21(4):755-65.

18. Benesh AE, Fleming JT, Chiang C, Carter BD, Tyska MJ. Expression and localization of myosin-1d in the developing nervous system. Brain Res. 2012 Feb 27;1440:9-22.

19. Lintas C, Sacco R, Tabolacci C, et al. An Interstitial 17q11.2 de novo Deletion Involving the CDK5R1 Gene in a High-Functioning Autistic Patient. Mol Syndromol. 2019 Jan;9(5):247-52.

20. Fitzgerald J, Kennedy D, Viseshakul N, et al. UNCL, the mammalian homologue of UNC-50, is an inner nuclear membrane RNA-binding protein. Brain Res. 2000 Sep 15;877(1):110-23.

21. Abiusi E, D'Alessandro M, Dieterich K, et al. Biallelic mutation of UNC50, encoding a protein involved in AChR trafficking, is responsible for arthrogryposis. Hum Mol Genet. 2017 Oct 15;26(20):3989-94.

22. Steinlein O, Weiland S, Stoodt J, Propping P. Exon-intron structure of the human neuronal nicotinic acetylcholine receptor alpha 4 subunit (CHRNA4). Genomics. 1996 Mar 1;32(2):289-94.

23. De Fusco M, Becchetti A, Patrignani A, et al. The nicotinic receptor beta 2 subunit is mutant in nocturnal frontal lobe epilepsy. Nat Genet. 2000 Nov;26(3):275-6.

24. Aridon P, Marini C, Di Resta C, et al. Increased sensitivity of the neuronal nicotinic receptor alpha 2 subunit causes familial epilepsy with nocturnal wandering and ictal fear. Am J Hum Genet. 2006 Aug;79(2):342-50.

25. Comartin D, Gupta GD, Fussner E, et al. CEP120 and SPICE1 cooperate with CPAP in centriole elongation. Curr Biol. 2013 Jul 22;23(14):1360-6.

26. Maes T, Barcelo A, Buesa C. Neuron navigator: a human gene family with homology to unc-53, a cell guidance gene from Caenorhabditis elegans. Genomics. 2002 Jul;80(1):21-30.

27. Coy JF, Wiemann S, Bechmann I, et al. Pore membrane and/or filament interacting like protein 1 (POMFIL1) is predominantly expressed in the nervous system and encodes different protein isoforms. Gene. 2002 May 15;290(1-2):73-94.

28. Pederick DT, Richards KL, Piltz SG, et al. Abnormal Cell Sorting Underlies the Unique X-Linked Inheritance of PCDH19 Epilepsy. Neuron. 2018 Jan 3;97(1):59-66 e5.

29. Li HJ, Haque ZK, Chen A, Mendelsohn M. RIF-1, a novel nuclear receptor corepressor that associates with the nuclear matrix. J Cell Biochem. 2007 Nov 1;102(4):1021-35.

30. Lu B, Zhang Q, Wang H, Wang Y, Nakayama M, Ren D. Extracellular calcium controls background current and neuronal excitability via an UNC79-UNC80-NALCN cation channel complex. Neuron. 2010 Nov 4;68(3):488-99.

31. Chong JX, McMillin MJ, Shively KM, et al. De novo mutations in NALCN cause a syndrome characterized by congenital contractures of the limbs and face, hypotonia, and developmental delay. Am J Hum Genet. 2015 Mar 5;96(3):462-73.

32. Al-Sayed MD, Al-Zaidan H, Albakheet A, et al. Mutations in NALCN cause an autosomal-recessive syndrome with severe hypotonia, speech impairment, and cognitive delay. Am J Hum Genet. 2013 Oct 3;93(4):721-6.

33. Shamseldin HE, Faqeih E, Alasmari A, Zaki MS, Gleeson JG, Alkuraya FS. Mutations in UNC80, Encoding Part of the UNC79-UNC80-NALCN Channel Complex, Cause Autosomal-Recessive Severe Infantile Encephalopathy. Am J Hum Genet. 2016 Jan 7;98(1):210-5.

34. Stray-Pedersen A, Cobben JM, Prescott TE, et al. Biallelic Mutations in UNC80 Cause Persistent Hypotonia, Encephalopathy, Growth Retardation, and Severe Intellectual Disability. Am J Hum Genet. 2016 Jan 7;98(1):202-9.

35. Lu B, Su Y, Das S, Liu J, Xia J, Ren D. The neuronal channel NALCN contributes resting sodium permeability and is required for normal respiratory rhythm. Cell. 2007 Apr 20;129(2):371-83.

36. Li W, Shen W, Zhang B, et al. Long non-coding RNA LncKdm2b regulates cortical neuronal differentiation by cis-activating Kdm2b. Protein Cell. 2020 Mar;11(3):161-86.

37. Bachmann-Gagescu R, Phelps IG, Dempsey JC, et al. KIAA0586 is Mutated in Joubert Syndrome. Hum Mutat. 2015 Sep;36(9):831-5.

38. Alby C, Piquand K, Huber C, et al. Mutations in KIAA0586 Cause Lethal Ciliopathies Ranging from a Hydrolethalus Phenotype to Short-Rib Polydactyly Syndrome. Am J Hum Genet. 2015 Aug 6;97(2):311-8.

39. Pauli S, Altmuller J, Schroder S, et al. Homozygosity for the c.428delG variant in KIAA0586 in a healthy individual: implications for molecular testing in patients with Joubert syndrome. J Med Genet. 2019 Apr;56(4):261-4.

40. Samocha KE, Robinson EB, Sanders SJ, et al. A framework for the interpretation of de novo mutation in human disease. Nat Genet. 2014 Sep;46(9):944-50.

41. Lek M, Karczewski KJ, Minikel EV, et al. Analysis of protein-coding genetic variation in 60,706 humans. Nature. 2016 Aug 18;536(7616):285-91.

42. Havrilla JM, Pedersen BS, Layer RM, Quinlan AR. A map of constrained coding regions in the human genome. Nat Genet. 2019 Jan;51(1):88-95.

43. Kircher M, Witten DM, Jain P, O'Roak BJ, Cooper GM, Shendure J. A general framework for estimating the relative pathogenicity of human genetic variants. Nat Genet. 2014 Mar;46(3):310-5.

44. Team RC. R: A Language and Environment for Statistical Computing. R Foundation for Statistical Computing; 2013; Available from: <http://www.R-project.org/>.

| ***Supplementary Table 1.*** Clinical characteristics in individuals with FIRES | | | | | | | | | | | |  |  |
| --- | --- | --- | --- | --- | --- | --- | --- | --- | --- | --- | --- | --- | --- |
|  | **Pat. no.** | **Sex** | **Development before seizure onset** | **Age at onset (years)** | **Prodromal phase** | **Acute epileptic phase: seizure type and duration** | **EEG at acute phase** | **MRI at onset: first week / during course** | **Chronic epileptic phase: seizure types and frequency** | **Outcome including level of impairment** | **Intellectual disability** | **Age at last follow-up (years)** |  |
|  | F1 | F | Normal | 6 | Febrile rhinovirus bronchitis | Focal and GTC SE (12w) | Bitemporal discharges | Bilateral T_2_ hyperintensities of insulae, amygdalae, and hippocampi / cerebral atrophy | Focal and myoclonic (daily) | MCS | Severe | 17 |  |
|  | F3 | F | 2 FS | 6 | Febrile upper respiratory infection | Focal and GTC SE (4w) | Multifocal discharges | Slight bilateral T_2_ hyperintensities of insulae and hippocampi / bilateral hippocampal atrophy | GTC (daily) | MCS | Severe | 24 |  |
|  | F4 | M | Normal | 3 | Febrile upper respiratory infection | Tonic SE (8w) | Diffuse slowing, bifrontal discharges | Normal / cerebral atrophy | Focal and tonic (daily) | MCS; deceased (11y) | Severe | 11 (deceased) |  |
|  | F5 | F | Normal | 6 | Subfebrile temperature, vomiting, headache | Focal and GTC SE (4w) | Diffuse slowing | Normal / mild bilateral hippocampal sclerosis | Focal (daily) | Learning deficit, speech delay, aphasia | Moderate | 12 |  |
|  | F6 | F | Normal | 2 | Rhinitis with slight fever | Focal (eyes deviation, cyanosis) with  generalization (5w) | Poor background organization,  focal temporal-frontal discharges | Normal with mild lateral ventricles enlargement / stable with no additional anomalies | Hemiclonic seizures (5-6/y) | Visuospatial deficit and behavior disturbances | Mild | 12 |  |
|  | F7 | M | Normal | 3 | Febrile pneumonia | Focal and GTC SE (1w) | Diffuse slowing, temporal discharges left-sided | Slight T_2_ hyperintensities of left insula and hippocampi / normal | Focal (monthly clusters) | Mild speech delay | Mild | 7 |  |
|  | F8 | M | Normal | 12 | Fever and headache | Focal and GTC SE (4w) | Bitemporal discharges | Normal / mild cerebral atrophy | Focal and GTC (weekly cluster) | Cognitive and learning deficits; deceased (29y) | Severe | 21 (deceased) |  |
|  | F9 | F | Normal | 6 | Febrile tonsillitis | Focal clonic, bilateral (3w) | Bitemporal and bifrontal discharges | Normal / normal | 5/m | Executive function disorders, attention disorder, learning difficulties | Mild | 9 |  |
|  | F10 | M | Normal | 15 | Fever and headache | Focal, myoclonic, and nonconvulsive SE (3w) | Diffuse slowing | Normal / normal | GTC (infrequent) | Attention and memory deficits | No | 25 |  |
|  | F11 | M | Anxiety and obsessive-compulsive disorder (7y) | 11 | Febrile pharyngitis | Focal and GTC SE (5w) | Multifocal discharges | Diffuse edema and basal ganglia injury / cerebral atrophy | Tonic (daily) | MCS | Severe | 16 |  |
|  | F12 | M | Normal | 11 | Febrile cervical lymphadenitis | Tonic, clonic, and GTC SE (20w) | Diffuse slowing, bifrontal discharges | Hydrocephalus, cortical edema, basal ganglia injury / cerebral atrophy | Multifocal (daily) | MCS | Severe | 20 |  |
|  | F13 | F | Normal | 7 | Febrile enteritis | Focal and GTC SE (2w) | Diffuse slowing with bifrontal discharges | Normal / normal | Focal (infrequent) | Verbal learning and memory deficits | No | 10 |  |
|  | F14 | M | Normal, ADHD | 6 | Febrile enteritis | Focal bilateral with GTC (6w) | Slow background activity, multifocal discharges | Bilateral hippocampal T2 hyperintensities / bilateral hippocampal atrophy | Focal (weekly-monthly) | Attention deficit, learning deficit | Mild | 14 |  |
|  | F15 | M | Normal | 2 | URT infection with fever | Eye and head deviation, focal (4w) | Slow background activity, bilateral sharp wave discharges | Normal / normal | Focal (daily) | Learning deficit | Moderate | 14 |  |
|  | F16 | M | Normal | 3 | Febrile tonsillitis | Focal bilateral with clonic seizures (3w) | Diffuse slowing with bifrontal and bitemporal discharges | Bitemporal T2 hyperintensities more to the left / bilateral hippocampal atrophy | Focal (6-8/w) | Frontal lobe syndrome, memory deficit,  learning deficit | Moderate | 8 |  |
|  | F17 | M | Juvenile oligoarthritis | 5 | Fever | Focal and GTC SE (1.5w) | Diffuse slowing, focal discharges | Normal / bilateral T2-hyperintensities of claustrum | Complex-focal (weekly) | Memory and speech deficits | Mild | NA |  |

|  | F18 | M | Normal | 8 | Febrile angina, headache, and knee pain | Focal SE (5w) | | Diffuse slowing, focal discharges | | Normal / bilateral hippocampal atrophy | | Multifocal with temporal predilection (nearly daily) | Memory and learning deficits | Moderate | 14 |  |
| --- | --- | --- | --- | --- | --- | --- | --- | --- | --- | --- | --- | --- | --- | --- | --- | --- |
|  | F19 | F | Normal | 4 | Febrile pharyngitis | | Focal and myoclonic SE (1.5w) | Focal discharges | Bilateral T_2_ hyperintensities of external capsule and extrema / cerebral atrophy | | Rolandic (infrequently on AED) | | Motor deficit, visual impairment | Moderate | 16 |  |
|  | F20 | M | Normal | 6 | Fever, headache, and gastrointestin-al symptoms | | Focal and GTC SE (1.5w) | Diffuse slowing, multifocal discharges | Normal / normal | | Focal and sec. GTC (weekly) | | Cognitive deficit | Moderate | 12 |  |
|  | F21 | M | Normal | 7 | Febrile RSV-bronchopneu-monia | | Recurrent focal and GTC (2w) | Diffuse slowing, focal discharges | CCT normal / cerebral atrophy | | Complex-focal and sec. GTC (weekly) | | Nystagmus, speech deficit, ataxia after seizures, behavioral disturbances | Mild to moderate | 17 |  |
|  | F22 | M | Normal | 8 | Fever, gastrointestin-al symptoms, and headache | | Myoclonic and tonic SE (2w) | Diffuse slowing, multifocal discharges | Normal / bilateral hippocampal sclerosis | | Cluster of focal and sec. GTC (weekly to monthly) | | Learning and memory deficits, behavioral disturbances | Moderate | 19 |  |
|  | F23 | M | Normal | 6 | Febrile angina | | Focal and GTC SE (5m) | Bitemporal discharges | Cerebral atrophy / cerebral atrophy | | Rolandic (daily) | | Disabled; deceased (8y) | Severe | 8 (deceased) |  |
|  | F24 | M | Normal motor development, support with schooling | 7 | Fever with rhinitis | | Focal with SE (L or R focal motor, tonic, chewing and complex automatisms) (4w) | Theta waves in R parietal region, slow background | Bilateral T_2_ and FLAIR peri-insular and temporo-mesial hyperintensities right-sided / normal | | Focal (monthly),  (left hand paresthesia, left eye and head deviation) | | Visuo-spatial disturbances | No | 30 |  |
|  | F25 | M | Normal | 11 | URT Infection with fever | | Focal rapidly bilateral with TC seizures | Bilateral frontal delta and theta waves with temporal and frontal discharges | Normal / slight periventricular hyperintensities | | Seizure clusters (monthly), visual hallucination, and paresthesia | | Fine motor impairment | Mild | 18 |  |

|  | F26 | F | Normal | 4 | Fever | GTC SE (2w) | Burst suppression, bifrontal discharges | Normal / cerebral atrophy | GTC (weekly) | Autism, axial, oral hypotonia | Moderate | 6 |  |
| --- | --- | --- | --- | --- | --- | --- | --- | --- | --- | --- | --- | --- | --- |
|  | F27 | M | Normal | 7 | Fever | Focal and GTC SE (2w), cyanosis, and eye deviation (5w) | Poor background activity | Bilateral T_2_, FLAIR, DWI frontal, parietal, temporal, and insular hyperintensities (insular atrophy) / no data | Seizure free under AEDs | Memory and behavior disorders | Moderate | 25 |  |
|  | F28 | M | Normal | 6 | Febrile bronchitis | Focal, hemiclonic, bilateral cyanosis (2w) | Temporal spikes, delta waves | Bilateral T_2_ and FLAIR insular hyperintensities / hippocampus and insula atrophy | Focal seizures with motor arrest and focal head deviation (daily) | Speech and memory disorders with fine motor impairment hippocampus | Moderate | 23 |  |
|  | F29 | M | Normal | 4 | Influenza | Complex-focal SE (1w) | Diffuse slowing, bilateral discharges | Normal / normal | Complex-focal seizures (daily) | Behavioral disturbances | Moderate | 8 |  |
|  | F30 | F | NA | 6 | Febrile tonsillitis | Complex-focal and sec. GTC SE (15min) and clusters | Diffuse slowing, bilateral discharges | Normal / normal | GTC (weekly) | Behavioral disturbances | Moderate | 13 |  |
|  | F31 | M | Normal | 5 | Fever and gastrointestin-al symptoms | GTC SE (3w) | Diffuse slowing, multifocal discharges | T2 hyperintensities of brainstem and cerebellum right-sided / cerebral atrophy | Complex-focal (weekly to monthly) | Autism, deficits | Mild | 7 |  |
|  | F32 | F | Normal | 7 | Influenza | Complex-focal SE (3w) | Diffuse slowing | Minimal cerebellar atrophy, hippocampal sclerosis / normal | Complex-focal and sec. GTC (daily) | Language and short-term memory deficits | Mild | 9 |  |
|  | F33 | M | Normal | 8 | Influenza | Eye deviation, hemiclonic jerks, cyanosis, and SE (2.5w) | Poor background activity | Bilateral DWI and FLAIR insular hyperintensities / hippocampal atrophy | Tonic and atonic seizures, eye and head deviation, rare sec. GTC (daily-weekly) | Motor and communication disorders (Autism spectrum disorder) | Severe | 30 |  |

|  | F34 | M | Normal | 2 | Fever | Head deviation, oral automatisms, hemifacial jerks, GTC SE (4w) | Bilateral temporal epileptiform abnormalities, sleep activation | Normal / cerebellar atrophy and bilateral hippocampal hyperintensities | Seizures secondarily generalized (daily-weekly) | Motor disorder, hyperactivity | Moderate | 13 |  |
| --- | --- | --- | --- | --- | --- | --- | --- | --- | --- | --- | --- | --- | --- |
|  | F35 | F | Normal | 5 | Febrile infection | Simple partial, complex partial seizures (2m) | Multifocal bilateral | Normal / cerebral atrophy | No data | Partial recovery | Severe | 14 |  |
|  | F36 | M | Normal | 4 | Febrile infection | Simple partial seizures, secondary generalized (1w) | Bilateral | Mild cerebral atrophy / no data | No data | Partial recovery | Borderline | 10 |  |
|  | F37 | M | Normal | 12 | Febrile infection | Simple partial seizures (1m) | Multifocal bilateral | Normal / no data | No data | Learning and attention deficits | No | 24 |  |
|  | F38 | F | Normal | 8 | Febrile infection | Simple partial seizures, secondary generalized (3w) | Diffuse slowing | Ependymal enhancement / no data | No data | Partial recovery | Borderline | NA |  |
|  | F39 | M | Normal | 11 | Febrile infection | Facial twitching, simple partial seizures, secondary generalized (6.5w) | Bilateral | Normal / normal | No data | Partial recovery | Moderate | NA |  |
|  | F40 | M | Normal | 5 | Febrile infection | Simple partial seizures, secondary generalized  (no data) | Multifocal bilateral | External capsule enhancement / normal | No data | Partial recovery | No data | 6 |  |
|  | F41 | M | Normal | 9 | Febrile infection | Facial twitching, simple partial seizures, secondary generalized (1m) | Multifocal bilateral | Normal / cerebral atrophy | No data | Partial recovery | Moderate | NA |  |
|  | F42 | F | Normal | 6 | Febrile tonsillitis | Focal, myoclonic, and GTC SE (11w) | Diffuse slowing, multifocal discharges | Normal / cerebral atrophy | Focal (daily) | MCS | Severe | 13 |  |

|  | F43 | M | Language delay (stutter) | 6 | Febrile *Parvovirus B19* infection | Focal and GTC SE (1w) | Frontotemporal discharges left-sided with generalization | Bilateral T_2_ hyperintensities of insulae and temporal lobes | Focal and sec. GTC (monthly cluster) | Attention deficit, behavioral disturbances | Mild | 20 |  |
| --- | --- | --- | --- | --- | --- | --- | --- | --- | --- | --- | --- | --- | --- |
|  | F44 | F | 3 Febrile SE | 14 | Febrile enteritis | Focal and GTC SE (1w) | Diffuse slowing, multifocal discharges | Normal / normal | No seizures on AEDs | Normal | No | 14 |  |
|  | F45 | F | Normal | 3 | Febrile upper respiratory infection | Focal, myoclonic, and GTC SE (7w) | Diffuse slowing, multifocal discharges | Posterior stroke left-sided, restricted diffusion in basal ganglia, cerebral atrophy | Stable on dextro-methorphan | MCS, spastic tetraplegia, deceased (3y) | Severe | 3 (deceased) |  |
|  | F46 | M | Normal | 9 | Fever | Focal (tonic-clonic) and GTC SE (2w) | Diffuse slowing | Normal / normal | Focal and sec. GTC (daily) | Cognitive deficit, behavioral disturbances | Mild | 14 |  |
|  | F47 | M | Normal | 13 | Febrile pharyngitis and upper respiratory infection | GTC SE (2w) | Multifocal epileptic activity | Normal / mild cerebral atrophy | Complex focal seizures (weekly) | Memory deficits | Mild | 22 |  |
|  | F48 | M | Normal | 5 | No data | GTC SE (no data) | Bitemporal discharges | Cerebral atrophy and hydrocephalus / no data | No data | Disabled | Severe | 6 |  |
|  | F49 | F | Normal | 5 | Febrile upper respiratory infection | SE of unreported type (6w) | Diffuse slowing, occipital discharges, PPR | Normal | No seizures on AEDs | Requires assistance in all aspects of life | Severe | 6 |  |
|  | F50 | M | Normal | 6 | Fever, headache, and neck pain | Focal SE (no data) | Multifocal discharges | Cerebral atrophy | Tonic seizures (daily) | Developmental regression, cortical visual impairment | Moderate | 7 |  |
|  | F51 | F | 2 FS | 2 | Fever, gastroenteritis,  urinary tract infection | Febrile SE (>60 min) | Diffuse slowing, bitemporal discharges | Normal / cerebral atrophy, T_2_ hyperintensities | Atypical absence, tonic, atonic seizures (20/day) | Apraxia, dystonia, developmental delays | Moderate | 5 |  |
|  | Abbreviations: AED, antiepileptic drug; CCT, cranial computer tomography; EEG, electroencephalography; F, female; GTC(S), generalized tonic-clonic (seizure); FS, Febrile seizure(s); M, male; m, month(s); MCS, minimally conscious state; MRI, magnetic resonance imaging; OCD, obsessive compulsive disorder; PPR, positive photoparoxysmal reaction; SE, status epilepticus; sec, secondary; URT, upper respiratory tract; w, week(s); y, years(s) | | | | | | | | | | | |  |

|  | ***Supplementary Table 2*.** Gene list for virtual epilepsy gene panel | | | |  |
| --- | --- | --- | --- | --- | --- |
|  | **Gene** | **Associated disease** | **OMIM ID** | **Inheritance** |  |
|  | *ALDH7A1* | Epilepsy, pyridoxine-dependent | 107323 | AR |  |
|  | *ALG13* | Congenital disorder of glycosylation, Early infantile epileptic encephalopathy | 300776 | XLD |  |
|  | *ARHGEF9* | Early infantile epileptic encephalopathy | 300429 | XLR |  |
|  | *ARX* | Early infantile epileptic encephalopathy, Hydranencephaly with abnormal genitalia, Lissencephaly, Mental retardation, Partington syndrome, Proud syndrome, | 300382 | XL/XLR |  |
|  | *ASAH1* | Farber lipogranulomatosis, Spinal muscular atrophy with progressive myoclonic epilepsy | 613468 | AR |  |
|  | *CACNA1A* | Early infantile epileptic encephalopathy, Spinocerebellar ataxia, Familial hemiplegic migraine, Episodic ataxia | 601011 | AD |  |
|  | *CACNA1E* | Early infantile epileptic encephalopathy | 601013 | AD |  |
|  | *CACNA1G* | Spinocerebellar ataxia, early-onset, severe, with neurodevelopmental deficits | 604065 | AD |  |
|  | *CASK* | FG syndrome 4, Mental retardation and microcephaly with pontine and cerebellar hypoplasia, Mental retardation, with or without nystagmus | 300172 | XLD |  |
|  | *CDKL5* | Early infantile epileptic encephalopathy | 300203 | XLD |  |
|  | *CHD2* | Epileptic encephalopathy, childhood-onset | 602119 | AD |  |
|  | *CHRNA4* | Epilepsy, nocturnal frontal lobe | 118504 | AD |  |
|  | *CHRNB2* | Epilepsy, nocturnal frontal lobe | 118507 |  |  |
|  | *CLN3* | Ceroid lipofuscinosis, neuronal | 607042 | AR |  |
|  | *CLN5* | Ceroid lipofuscinosis, neuronal | 608102 | AR |  |
|  | *CLN6* | Ceroid lipofuscinosis, neuronal, Ceroid lipofuscinosis, neuronal, Kufs type, adult onset | 606725 | AR |  |
|  | *CLN8* | Ceroid lipofuscinosis, neuronal, Ceroid lipofuscinosis, neuronal, 8, Northern epilepsy variant | 607837 | AR |  |
|  | *CSTB* | Progressive myoclonic epilepsy | 601145 | AR |  |
|  | *CTSD* | Ceroid lipofuscinosis, neuronal | 116840 | AR |  |
|  | *DEPDC5* | Epilepsy familial focal with variable foci | 614191 | AD |  |
|  | *DNM1* | Early infantile epileptic encephalopathy | 602377 | AD |  |
|  | *DYNC1H1* | Spinal muscular atrophy, lower extremity-predominant, Intellectual disability | 600112 | AD |  |
|  | *DYRK1A* | Mental retardation | 600855 | AD |  |
|  | *EEF1A2* | Early infantile epileptic encephalopathy | 602959 | AD |  |
|  | *EPM2A* | Epilepsy, progressive myoclonic 2A (Lafora) | 607566 | AR |  |
|  | *FOLR1* | Neurodegeneration due to cerebral folate transport deficiency | 136430 | AR |  |
|  | *FOXG1* | Rett syndrome, congenital variant | 164874 | AD |  |
|  | *GABRA1* | Early infantile epileptic encephalopathy | 137160 | AD |  |
|  | *GABRB3* | Early infantile epileptic encephalopathy | 137192 | AD |  |
|  | *GABRG2* | Epilepsy, generalized, with febrile seizures plus type 3, Febrile seizures familial, Early infantile epileptic encephalopathy | 137164 | AD |  |
|  | *GAMT* | Cerebral creatine deficiency syndrome 2 | 601240 | AR |  |
|  | *GATM* | Cerebral creatine deficiency syndrome 3 | 602360 | AR |  |
|  | *GNAO1* | Early infantile epileptic encephalopathy, Neurodevelopmental disorder with involuntary movements | 139311 | AD |  |
|  | *GRIN1* | Neurodevelopmental disorder with or without hyperkinetic movements and seizures | 138249 | AD/AR |  |
|  | *GRIN2A* | Epilepsy, focal, with speech disorder and with or without intellectual disability | 138253 | AD |  |
|  | *GRIN2B* | Early infantile epileptic encephalopathy, Intellectual disability | 138252 | AD |  |
|  | *GRIN2D* | Early infantile epileptic encephalopathy | 602717 | AD |  |
|  | *HNRNPU* | Early infantile epileptic encephalopathy | 602869 | AD |  |
|  | *IQSEC2* | Intellectual disability | 300522 | XLD |  |
|  | *KCNA1* | Episodic ataxia/myokymia syndrome | 176260 | AD |  |
|  | *KCNA2* | Early infantile epileptic encephalopathy | 176262 | AD |  |
|  | *KCNB1* | Early infantile epileptic encephalopathy | 600397 | AD |  |
|  | *KCNC1* | Epilepsy, progressive myoclonic 7 | 176258 | AD |  |
|  | *KCNJ10* | Enlarged vestibular aqueduct digenic, SESAME syndrome | 602208 | AR |  |
|  | *KCNQ2* | Benign neonatal seizures, myokymia, Early infantile epileptic encephalopathy | 602235 | AD |  |
|  | *KCNQ3* | Seizures, benign neonatal 2 | 602232 | AD |  |
|  | *KCNQ5* | Mental retardation | 607357 | AD |  |
|  | *KCNT1* | Nocturnal frontal lobe epilepsy, Early infantile epileptic encephalopathy | 608167 | AD |  |
|  | *KCTD7* | Epilepsy, progressive myoclonic 3, with or without intracellular inclusions | 611725 | AR |  |
|  | *LGI1* | Epilepsy, familial temporal lobe | 604619 | AD |  |
|  | *MECP2* | Neonatal severe encephalopathy, Rett syndrome | 300005 | XLR/XLD |  |
|  | *MEF2C* | Chromosome 5q14.3 deletion syndrome, Mental retardation, stereotypic movements, epilepsy, and/or cerebral malformations | 600662 | AD |  |
|  | *MFSD8* | Ceroid lipofuscinosis, neuronal 7, Macular dystrophy with central cone involvement | 611124 | AR |  |
|  | *MTOR* | Focal cortical dysplasia type II, Smith-Kingsmore syndrome | 601231 | AD |  |
|  | *NEXMIF* | Intellectual disability | 300524 | XLD |  |
|  | *NHLRC1* | Epilepsy, progressive myoclonic 2B (Lafora) | 608072 | AR |  |
|  | *NPRL2* | Epilepsy, familial focal, with variable foci 2 | 607072 | AD |  |
|  | *NPRL3* | Epilepsy, familial focal, with variable foci 3 | 600928 | AD |  |
|  | *PCDH19* | Early infantile epileptic encephalopathy | 300460 | XL |  |
|  | *PIGA* | Multiple congenital anomalies-hypotonia-seizures syndrome 2 | 311770 | XLR |  |
|  | *PIGO* | Hyperphosphatasia with mental retardation syndrome 2 | 614730 | AR |  |
|  | *PIGT* | Multiple congenital anomalies-hypotonia-seizures syndrome 3, Paroxysmal nocturnal hemoglobinuria 2 Somatic mutation | 610272 | AD/AR |  |
|  | *PLCB1* | Early infantile epileptic encephalopathy | 607120 | AR |  |
|  | *PNKP* | Ataxia-oculomotor apraxia 4, Microcephaly, seizures, and developmental delay | 605610 | AR |  |
|  | *PNPO* | Pyridoxamine 5'-phosphate oxidase deficiency | 603287 | AR |  |
|  | *POLG* | Mitochondrial DNA depletion syndrome 4A (Alpers type), Mitochondrial DNA depletion syndrome 4B (MNGIE type), Progressive external ophthalmoplegia, Mitochondrial recessive ataxia syndrome (includes SANDO and SCAE), Progressive external ophthalmoplegia | 174763 | AR/AD |  |
|  | *PPT1* | Ceroid lipofuscinosis, neuronal, 1 | 600722 | AR |  |
|  | *PRICKLE1* | Epilepsy, progressive myoclonic 1B | 608500 | AR |  |
|  | *PRRT2* | Convulsions familial infantile, with paroxysmal choreoathetosis, Episodic kinesigenic dyskinesia, Benign familial infantile seizures | 614386 | AD |  |
|  | *PURA* | Intellectual disability | 600473 | AD |  |
|  | *QARS1* | Microcephaly, progressive, seizures, and cerebral and cerebellar atrophy | 603727 | AR |  |
|  | *RELN* | Epilepsy, familial temporal lobe 7, Lissencephaly 2 | 600514 | AD/AR |  |
|  | *SCARB2* | Epilepsy, progressive myoclonic with or without renal failure | 602257 | AR |  |
|  | *SCN1A* | Epilepsy generalized with febrile seizures plus | 182389 | AD |  |
|  | *SCN1B* | Atrial fibrillation, familial, Brugada syndrome 5, Cardiac conduction defect, nonspecific, Epilepsy, generalized, with febrile seizures plus type 1, Epileptic encephalopathy, early infantile | 600235 | AD/AR |  |
|  | *SCN2A* | Benign familial infantile seizures, Early infantile epileptic encephalopathy | 182390 | AD |  |
|  | *SCN3A* | Epilepsy, familial focal with variable foci 4, Early infantile epileptic encephalopathy | 182391 | AD |  |
|  | *SCN8A* | Cognitive impairment with or without cerebellar ataxia, Early infantile epileptic encephalopathy, Benign familial infantile seizures | 600702 | AD |  |
|  | *SLC12A5* | Idiopathic generalized epilepsy, Early infantile epileptic encephalopathy | 606726 | AD/AR |  |
|  | *SLC13A5* | Idiopathic generalized epilepsy, Early infantile epileptic encephalopathy | 608305 | AR |  |
|  | *SLC25A22* | Early infantile epileptic encephalopathy | 609302 | AR |  |
|  | *SLC2A1* | Idiopathic generalized epilepsy, Dystonia, Stomatin-deficient cryohydrocytosis with neurologic defects, GLUT1 deficiency syndrome | 138140 | AD |  |
|  | *SLC35A2* | Congenital disorder of glycosylation type II | 314375 | XLD |  |
|  | *SLC6A1* | Myoclonic-atonic epilepsy | 137165 | AD |  |
|  | *SLC6A8* | Cerebral creatine deficiency syndrome | 300036 | XLR |  |
|  | *SMC1A* | Cornelia de Lange syndrome 2 | 300040 | XLD |  |
|  | *SPTAN1* | Early infantile epileptic encephalopathy | 182810 | AD |  |
|  | *ST3GAL3* | Early infantile epileptic encephalopathy, Mental retardation | 606494 | AR |  |
|  | *STX1B* | Generalized epilepsy with febrile seizures plus | 601485 | AD |  |
|  | *STXBP1* | Early infantile epileptic encephalopathy | 602926 | AD |  |
|  | *SYN1* | Epilepsy with variable learning disabilities and behavior disorders | 313440 | XLD/XLR |  |
|  | *SYNGAP1* | Intellectual disability | 603384 | AD |  |
|  | *SZT2* | Early infantile epileptic encephalopathy | 615463 | AR |  |
|  | *TBC1D24* | Myoclonic epilepsy, DOORS syndrome (deafness, onychodystrophy, osteodystrophy, mental retardation and seizures), Early infantile epileptic encephalopathy | 613577 | AD/AR |  |
|  | *TPP1* | Ceroid lipofuscinosis, neuronal, Spinocerebellar ataxia | 607998 | AR |  |
|  | *TSC1* | Focal cortical dysplasia type II, Lymphangioleiomyomatosis, Tuberous sclerosis-1 | 605284 | AD |  |
|  | *TSC2* | Focal cortical dysplasia type II, Lymphangioleiomyomatosis, Tuberous sclerosis-2 | 191092 | AD |  |
|  | *UBE3A* | Angelman syndrome | 601623 | AD |  |
|  | *WDR45* | Neurodegeneration with brain iron accumulation 5 | 300526 | XLD |  |
|  | *WWOX* | Early infantile epileptic encephalopathy, Spinocerebellar ataxia | 605131 | AR |  |
|  | *ZEB2* | Mowat-Wilson syndrome | 605802 | AD |  |
|  | Abbreviations: AD, autosomal dominant; AR, autosomal recessive; XL, x-linked; XLD, x-linked dominant; XLR, x-linked recessive | | | |  |

|  | ***Supplementary Table 3*.** Comparison of virtual gene panel content to epilepsy gene panel studies in the literature | | | | |
| --- | --- | --- | --- | --- | --- |
|  | **Gene** | **Initial and added genes for virtual panel** | **Number of individuals with variants in gene panel studies^1^** | **Rank of gene amongst genes assessed in a least one gene panel study** |  |
|  | *SCN1A* | initial diagnostic panel | 742 | 1 |  |
|  | *KCNQ2* | initial diagnostic panel | 342 | 2 |  |
|  | *SCN2A* | initial diagnostic panel | 217 | 3 |  |
|  | *PRRT2* | initial diagnostic panel | 197 | 4 |  |
|  | *CDKL5* | initial diagnostic panel | 192 | 5 |  |
|  | *MECP2* | added to virtual panel | 185 | 6 |  |
|  | *PCDH19* | initial diagnostic panel | 162 | 7 |  |
|  | *STXBP1* | initial diagnostic panel | 158 | 8 |  |
|  | *POLG* | added to virtual panel | 106 | 9 |  |
|  | *SLC2A1* | initial diagnostic panel | 100 | 10 |  |
|  | *SCN8A* | initial diagnostic panel | 99 | 11 |  |
|  | *TSC2* | initial diagnostic panel | 88 | 12 |  |
|  | *DEPDC5* | initial diagnostic panel | 85 | 13 |  |
|  | *UBE3A* | initial diagnostic panel | 77 | 14 |  |
|  | *CACNA1A* | initial diagnostic panel | 76 | 15 |  |
|  | *TPP1* | initial diagnostic panel | 75 | 16 |  |
|  | *SYNGAP1* | initial diagnostic panel | 74 | 17 |  |
|  | *ALDH7A1* | initial diagnostic panel | 65 | 19 |  |
|  | *GRIN2A* | initial diagnostic panel | 63 | 20 |  |
|  | *KCNT1* | added to virtual panel | 63 | 21 |  |
|  | *GABRG2* | initial diagnostic panel | 62 | 22 |  |
|  | *FOXG1* | initial diagnostic panel | 54 | 23 |  |
|  | *CHD2* | initial diagnostic panel | 52 | 24 |  |
|  | *TSC1* | initial diagnostic panel | 51 | 25 |  |
|  | *GABRA1* | initial diagnostic panel | 47 | 26 |  |
|  | *TBC1D24* | initial diagnostic panel | 38 | 29 |  |
|  | *SPTAN1* | initial diagnostic panel | 36 | 31 |  |
|  | *ZEB2* | initial diagnostic panel | 36 | 32 |  |
|  | *ARX* | initial diagnostic panel | 31 | 33 |  |
|  | *CLN3* | initial diagnostic panel | 30 | 35 |  |
|  | *GRIN2B* | initial diagnostic panel | 30 | 36 |  |
|  | *PNKP* | initial diagnostic panel | 29 | 37 |  |
|  | *PPT1* | initial diagnostic panel | 28 | 39 |  |
|  | *IQSEC2* | initial diagnostic panel | 26 | 40 |  |
|  | *PIGO* | initial diagnostic panel | 26 | 41 |  |
|  | *SZT2* | initial diagnostic panel | 25 | 42 |  |
|  | *CLN5* | initial diagnostic panel | 23 | 44 |  |
|  | *GABRB3* | initial diagnostic panel | 22 | 45 |  |
|  | *DYRK1A* | added to virtual panel | 21 | 47 |  |
|  | *HNRNPU* | initial diagnostic panel | 21 | 48 |  |
|  | *GAMT* | initial diagnostic panel | 20 | 54 |  |
|  | *SCN1B* | initial diagnostic panel | 20 | 56 |  |
|  | *ALG13* | initial diagnostic panel | 19 | 57 |  |
|  | *SMC1A* | added to virtual panel | 19 | 59 |  |
|  | *EPM2A* | initial diagnostic panel | 18 | 61 |  |
|  | *SLC6A1* | initial diagnostic panel | 18 | 62 |  |
|  | *MEF2C* | initial diagnostic panel | 17 | 64 |  |
|  | *NPRL3* | added to virtual panel | 17 | 65 |  |
|  | *PNPO* | initial diagnostic panel | 17 | 66 |  |
|  | *WWOX* | initial diagnostic panel | 17 | 67 |  |
|  | *CASK* | initial diagnostic panel | 16 | 68 |  |
|  | *KCNQ3* | initial diagnostic panel | 16 | 69 |  |
|  | *LGI1* | initial diagnostic panel | 15 | 74 |  |
|  | *NEXMIF* | added to virtual panel | 15 | 75 |  |
|  | *CHRNA4* | added to virtual panel | 13 | 79 |  |
|  | *CSTB* | initial diagnostic panel | 13 | 80 |  |
|  | *KCNB1* | initial diagnostic panel | 13 | 81 |  |
|  | *KCTD7* | initial diagnostic panel | 13 | 82 |  |
|  | *NHLRC1* | initial diagnostic panel | 13 | 84 |  |
|  | *PURA* | initial diagnostic panel | 13 | 87 |  |
|  | *CHRNB2* | added to virtual panel | 11 | 91 |  |
|  | *RELN* | initial diagnostic panel | 10 | 98 |  |
|  | *WDR45* | initial diagnostic panel | 10 | 99 |  |
|  | *ARHGEF9* | initial diagnostic panel | 9 | 101 |  |
|  | *EEF1A2* | initial diagnostic panel | 9 | 103 |  |
|  | *FOLR1* | initial diagnostic panel | 9 | 104 |  |
|  | *GNAO1* | initial diagnostic panel | 9 | 106 |  |
|  | *SLC13A5* | initial diagnostic panel | 9 | 110 |  |
|  | *SLC6A8* | initial diagnostic panel | 9 | 111 |  |
|  | *STX1B* | initial diagnostic panel | 9 | 112 |  |
|  | *SYN1* | initial diagnostic panel | 9 | 113 |  |
|  | *CLN6* | initial diagnostic panel | 8 | 119 |  |
|  | *DNM1* | initial diagnostic panel | 8 | 120 |  |
|  | *GRIN1* | initial diagnostic panel | 8 | 122 |  |
|  | *KCNA2* | initial diagnostic panel | 8 | 123 |  |
|  | *MFSD8* | initial diagnostic panel | 8 | 124 |  |
|  | *QARS* | initial diagnostic panel | 8 | 127 |  |
|  | *KCNJ10* | initial diagnostic panel | 7 | 138 |  |
|  | *PLCB1* | initial diagnostic panel | 7 | 139 |  |
|  | *SCN3A* | added to virtual panel | 7 | 140 |  |
|  | *MTOR* | added to virtual panel | 6 | 149 |  |
|  | *CLN8* | initial diagnostic panel | 5 | 159 |  |
|  | *KCNC1* | added to virtual panel | 5 | 167 |  |
|  | *SLC35A2* | initial diagnostic panel | 5 | 175 |  |
|  | *SLC12A5* | initial diagnostic panel | 4 | 202 |  |
|  | *SLC25A22* | initial diagnostic panel | 4 | 203 |  |
|  | *PRICKLE1* | initial diagnostic panel | 3 | 237 |  |
|  | *ST3GAL3* | initial diagnostic panel | 3 | 243 |  |
|  | *CTSD* | initial diagnostic panel | 2 | 258 |  |
|  | *GATM* | initial diagnostic panel | 2 | 270 |  |
|  | *SCARB2* | initial diagnostic panel | 2 | 298 |  |
|  | *ASAH1* | initial diagnostic panel | 1 | 319 |  |
|  | *DYNC1H1* | initial diagnostic panel | 1 | 343 |  |
|  | *GRIN2D* | added to virtual panel | 1 | 363 |  |
|  | *PIGA* | initial diagnostic panel | 1 | 398 |  |
|  | *KCNA1* | initial diagnostic panel | 0 | 574 |  |
|  | *CACNA1E* | added to virtual panel | not tested |  |  |
|  | *CACNA1G* | added to virtual panel | not tested |  |  |
|  | *KCNQ5* | added to virtual panel | not tested |  |  |
|  | *NPRL2* | added to virtual panel | not tested |  |  |
|  | *PIGT* | initial diagnostic panel | not tested |  |  |
|  | ^1^ Summarized variant data of three large gene panel studies in the epilepsy field, including 707 genes assessed in at least one study. The studies included were Lindy et al., 2018 (PMID 29655203, n=8565 individuals), Heyne et al., 2019 (PMID 31056551, n=6994 individuals), and Truty et al., 2019 (PMID 31440721, n=9769 individuals) | | | |  |

|  | ***Supplementary Table 4*.** Significant associations between HLA alleles and FIRES | | | | | | |
| --- | --- | --- | --- | --- | --- | --- | --- |
|  | **Allele** | **Freq case** | **Freq control** | **P uncorrected** | **OR [95% CI]** | **P FDR** |  |
|  | C*07:01 | 0.1379 | 0.0181 | 4.26x10^-5^ | 8.7 [3.55-21.30] | 7.23x10^-4^ |  |
|  | A*02:05 | 0.069 | 0.0057 | 0.0011 | 12.99 [3.56-47.39] | 0.0098 |  |
|  | A*03:01 | 0.0172 | 0.1427 | 0.0028 | 0.10 [0.01-0.77] | 0.0165 |  |
|  | Freq case=Frequency of HLA allele in cases; Freq control= Frequency of HLA allele in controls; P uncorr=P value for association without multiple testing correction; OR [95% CI]=odds ratio with 95% confidence interval; P FDR=multiple testing corrected P value using false discovery rate | | | | | |  |
